# Reduced Coefficients of Linear Thermal Expansion of Colorless and Transparent Semi-Alicyclic Polyimide Films via Incorporation of Rigid-Rod Amide Moiety: Preparation and Properties

**DOI:** 10.3390/polym12020413

**Published:** 2020-02-11

**Authors:** Gang-lan Jiang, Dong-yang Wang, Hao-peng Du, Xiao Wu, Yan Zhang, Yao-yao Tan, Lin Wu, Jin-gang Liu, Xiu-min Zhang

**Affiliations:** 1 Beijing Key Laboratory of Materials Utilization of Nonmetallic Minerals and Solid Wastes, National Laboratory of Mineral Materials, School of Materials Science and Technology, China University of Geosciences, Beijing 100083, China; 2003180029@cugb.edu.cn (G.-l.J.); 1003184104@cugb.edu.cn (H.-p.D.); 2103170022@cugb.edu.cn (X.W.); 2103170021@cugb.edu.cn (Y.Z.); 2003190022@cugb.edu.cn (Y.-y.T.); 2003190023@cugb.edu.cn (L.W.); 2 Beijing National Laboratory for Molecular Sciences, Key Laboratory of Organic Solids, Institute of Chemistry, Chinese Academy of Sciences, Beijing 100190, China; wangdongyang19@mails.ucas.ac.cn; 3 School of Electrical Engineering, Beijing Jiaotong University, Beijing 100044, China

**Keywords:** colorless polyimide film, coefficient of thermal expansion (CTE), amide, optical properties, thermal properties

## Abstract

Semi-alicyclic colorless and transparent polyimide (CPI) films usually suffer from the high linear coefficients of thermal expansion (CTEs) due to the intrinsic thermo-sensitive alicyclic segments in the polymers. A series of semi-alicyclic CPI films containing rigid-rod amide moieties were successfully prepared in the current work in order to reduce the CTEs of the CPI films while maintaining their original optical transparency and solution-processability. For this purpose, two alicyclic dianhydrides, hydrogenated pyromellitic anhydride (HPMDA, I), and hydrogenated 3,3’,4,4’-biphenyltetracarboxylic dianhydride (HBPDA, II) were polymerized with two amide-bridged aromatic diamines, 2-methyl-4,4’-diaminobenzanilide (MeDABA, a) and 2-chloro-4,4’-diaminobenzanilide (ClDABA, b) respectively to afford four CPI resins. The derived CPI resins were all soluble in polar aprotic solvents, including *N*-methyl-2-pyrrolidone (NMP) and *N*,*N*-dimethylacetamide (DMAc). Flexible and tough CPI films were successfully prepared by casing the PI solutions onto glass substrates followed by thermally cured at elevated temperatures from 80 °C to 250 °C. The MeDABA derived PI-I_a_ (HPMDA-MeDABA) and PI-II_a_ (HBPDA-MeDABA) exhibited superior optical transparency compared to those derived from ClDABA (PI-I_b_ and PI-II_b_). PI-I_a_ and PI-II_a_ showed the optical transmittances of 82.3% and 85.8% at the wavelength of 400 nm with a thickness around 25 μm, respectively. Introduction of rigid-rod amide moiety endowed the HPMDA-PI films good thermal stability at elevated temperatures with the CTE values of 33.4 × 10^−6^/K for PI-I_a_ and 27.7 × 10^−6^/K for PI-I_b_ in the temperature range of 50–250 °C. Comparatively, the HBPDA-PI films exhibited much higher CTE values. In addition, the HPMDA-PI films exhibited good thermal stability with the 5% weight loss temperatures (*T*_5%_) higher than 430 °C and glass transition temperatures (*T*_g_) in the range of 349–351 °C.

## 1. Introduction

The polymeric optical films with high thermal stability, high dimensional stability at elevated temperatures, high optical transparency, and high tensile properties are highly desired in modern optoelectronic areas, such as substrates or covering windows for flexible active matrix organic light-emitting diodes (AMOLED) devices, substrates for organic photovoltaic (OPV) solar cells, and so on [1,2,3]. Conventional polymeric optical films, such as polyolefin and polyester films usually suffer from the poor thermal and dimensional stability while the standard high-temperature resistant polymer films, such as the wholly aromatic polyimide (PI) films often exhibit highly colored appearance and poor optical transparency in the visible light region [4]. That is to say, the molecular structure factors that affect the optical and thermal properties of polymer films are usually contradictory. The structural factors that can improve the thermal stability of polymer films often reduce the optical properties of the films at the same time, and vice versa. Thus, it is a changeling research topic to develop polymer optical films with both of excellent thermal stability and good optical transparency [5,6,7].

In recent years, the research progress of colorless and transparent polyimide (CPI) films has attracted great attention in the research and development of high performance polymer optical films [8,9,10]. Based on the previous decades of research on the origination of coloration in traditional all-aromatic PI films, the researchers revealed many effective procedures to prohibit the formation of intra- or intermolecular charge transfer complexes (CTC) between the electron-donating diamine moiety and the electron-accepting dianhydride moiety so as to improve the optical transparency of the PI films [11,12,13]. At present, the effective means to develop CPI films mainly include introducing groups with high electronegativity characteristics, such as trifluoromethyl groups, sulphone groups, or substituents with non-conjugated characteristics, such as aliphatic or alicyclic groups, or groups with large molar volume—such as naphthalene, fluorene, or cardo groups—into the PI molecular chains [14,15,16,17,18,19,20]. Although various CPI films have been developed in the literature by these methodologies, much of them still cannot be practically used due to the absence of dimensional stability at elevated temperatures. High linear coefficients of thermal expansion (CTE) usually cause the mismatch of dimensional stability between the CPI components with the inorganic substrates, such as copper foil (CTE ≈ 17 × 10^−6^/K), silica wafer (CTE ≈ 3 × 10^−6^/K), and so on. Thus, severe reliability problems, such as delamination, cracking, warpage, and so on might occur in the practical application of CPI films in optoelectronic fabrications [21]. Recently, the development of solution-processable CPI films with ultra-low CTEs was systemically reviewed in the literature [22].

According to the chemical structures, the current CPI films mainly include the types of fluorine-containing all-aromatic one and the semi-alicyclic one. The former is mainly prepared by the two-step thermal imidization process with the fluorine-containing aromatic dianhydride, the rigid aromatic dianhydride, and fluorine-containing aromatic diamine monomers as the starting materials [23]. The use of rigid dianhydride monomer can usually endow the CPI films relatively low CTE values. Semi-alicyclic CPI films are usually prepared by the one-step high temperature imidization process or the two-step chemical imidization process by the polymerization of alicyclic dianhydride and aromatic diamine monomers. The CPI resin was first prepared by the above polymerization process, and then was dissolved in the corresponding solvent to form the soluble CPI solution. The CPI solution was thermally cured to remove the solvent at relatively low temperature to afford the CPI films. Compared with the fluorine-containing CPI films, the semi-alicyclic ones usually have the advantages of lower curing temperature and higher optical transmittance; however, higher CTE values [24]. Various efforts have been carried out in the literature to reduce the CTE values of semi-alicyclic CPI films, which can be characterized by the systematical and pioneering work reported by Hasegawa and coworkers [25,26,27,28,29]. Rigid ester bonds and amide bonds have been introduced into the molecular structures of the CPIs and the optical films with apparently reduced CTEs were successfully achieved. Among the rigid substituents that can efficiently reduce the CTE values of the CPI films, amide (–CONH–) bond seems to be one of the most promising species because it can usually endow the films high glass transition temperature (*T*_g_) via the formation of strong intra- or intermolecular hydrogen bonds in the polymer chains beside affording the polymers low-CTE features. However, the rigidity of the common amide-containing monomers, such as 4,4’-diaminobenzanilide (DABA) is usually too high to afford the CPI resins good solubility in organic solvents. Thus, the amide-containing PI films can only be prepared by the soluble poly(amic acid) (PAA) precursors followed by high-temperature imidization procedure [30,31].

In the current work, as one of our continuous work developing high performance CPI films for advanced optical applications, the reduction of CTE values of the semi-alicyclic CPI films were endeavored by introduction methyl substituents into the molecular structures of the amide-containing CPIs. For this purpose, a new amide-bridged diamine monomer, 2-methyl-4,4’-diamino-benzanilide (MeDABA) was first synthesized. Then, it was polymerized with the alicyclic dianhydrides, including hydrogenated pyromellitic anhydride (HPMDA) and hydrogenated 3,3’,4,4’-biphenyltetracarboxylic dianhydride (HBPDA), respectively. The effects of the amide units on the optical and thermal properties of the derived CPI films were investigated in detail.

## 2. Materials and Methods

### 2.1. Materials

Hydrogenated pyromellitic anhydride (HPMDA) and hydrogenated 3,3’,4,4’-biphenyltetracarboxylic dianhydride (HBPDA) were purchased from Weihai Newera Kesense New Materials Co. Ltd. (Shandong, China) and dried at 150 °C in vacuo for 24 h prior to use. 2-Chloro-4,4’-diaminobenzanilide (ClDABA) was purchased from Changzhou Sunlight Pharmaceutical Co. Ltd. (Jiangsu, China) and recrystallized from absolute ethanol and discolored with active charcoal powder before use. *N*-methyl-2-pyrrolidone (NMP), *N*,*N*-dimethylacetamide (DMAc), *N*,*N*-dimethylformamide (DMF), γ-butyrolactone (GBL), and other solvents were obtained from Tokyo Chemical Industry Co., Ltd. (Tokyo, Japan) and purified by distillation prior to use.

2-Methyl-4,4’-diaminobenzanilide (MeDABA) was synthesized in our laboratory according to the literature [32] and purified by recrystallization from absolute ethanol. MeDABA was obtained as colorless needles with the purity of 99.5% according to the gas chromatography analysis.

### 2.2. Characterization Methods

Absolute viscosity of the PI solutions was measured using a Brookfield DV-II+ Pro viscometer (Brookfield Ametek, Middleboro, MA, USA) with a cone spindle of CPA-41Z at 25 °C. The measurable viscosity limitation was 6–122,000 mPa s. Inherent viscosity of the PIs was measured using an Ubbelohde viscometer (Mitong Electromechanical Tech. Co. Ltd., Shanghai, China) with a 0.5 g dL^−1^ NMP solution at 25 °C. The number average molecular weight (*M*_n_) and weight average molecular weight (*M*_w_) of the SPI resins were measured using a gel permeation chromatography (GPC) system (Shimadzu, Kyoto, Japan) with a LC-20AD dual-plunger parallel-flow pumps (D1-LC), a SIL-20A is a total-volume injection-type auto-sampler, a CTO-20A column oven, and a RID-20A detector. HPLC grade NMP was used as the mobile phase at a flow rate of 1.0 mL/min. Attenuated total reflectance Fourier transform infrared (ATR-FTIR) spectra of the SPI ALs were recorded on a Iraffinity-1S FT-IR spectrometer (Shimadzu, Kyoto, Japan). Nuclear magnetic resonances (^1^H-NMR) were performed on an AV 400 spectrometer (Ettlingen, Germany) operating at 400 MHz in DMSO-*d*_6_. Ultraviolet–visible (UV–Vis) spectra were recorded on a Hitachi U-3210 spectrophotometer (Tokyo, Japan) at room temperature. Wide-angle X-ray diffraction (XRD) was conducted on a Rigaku D/max-2500 X-ray diffractometer (Tokyo, Japan) with Cu-Kα1 radiation, operated at 40 kV and 200 mA. Yellow index (YI) values of the PI films were measured using an X-rite color i7 spectrophotometer (Grand Rapids, MI, USA) with PI samples at a thickness of 50 µm in accordance with the procedure described in ASTM D1925 “Test Method for Yellowness Index of Plastics”. The color parameters were recorded according to a CIE Lab equation. L* is the lightness, where 100 means white and 0 implies black. A positive a* means a red color, and a negative one indicates a green color. A positive b* means a yellow color, and a negative one indicates a blue color. Thermogravimetric analysis (TGA) and differential scanning calorimetry (DSC) were recorded on a TA-Q series thermal analysis system (New Castle, DE, USA) at a heating rate of 10 °C/min in nitrogen. Dynamic mechanical analysis (DMA) was recorded on a TA-Q800 thermal analysis system (New Castle, DE, USA) at a heating rate of 5 °C/min and a frequency of 1 Hz in nitrogen.

Solubility was investigated by mixing 1.0 g of the PI resin and 9.0 g of the solvent tested (10 wt % solid content), and then stirred for 24 h at room temperature. The solubility was determined visually as three grades: completely soluble (++), partially soluble (+), and insoluble (−), wherein complete soluble indicates a homogenous and clean state without phase separation, precipitation or gel formation, and insoluble indicates no change of the resin in the appearance.

### 2.3. PI Synthesis and Film Preparation

The PI resins, including PI-I_a_ (HPMDA-MeDABA), PI-I_b_ (HPMDA-ClDABA), PI-II_a_ (HBPDA-MeDABA), and PI-II_b_ (HBPDA-ClDABA) were all synthesized via a one-step high-temperature polycondensation procedure, as can be illustrated by the synthesis of PI-I_a_. MeDABA (24.1290 g, 100 mmol) was added into the dried GBL (100.0 g) in a 500 mL three-necked flask equipped with a mechanical stirrer, a Dean–Stark trap and a nitrogen inlet. Clear diamine solution was obtained after stirring at room temperature for 10 min under the flow of nitrogen. Then, HPMDA (22.4170 g, 100 mmol) was added to the diamine solution together with an additional volume of GBL (39.6 g). The molar ratio of the HPMDA dianhydride and the MeDABA diamine was 1:1 and the solid content of the reaction system was controlled to be 25 wt %. Isoquinoline (0.5 g) was added as an imidization catalyst. After stirring in nitrogen for 1 h, toluene (150 mL) was then added into the flask as an azeotropic agent. The reaction mixture was heated to 180 °C and maintained for 6 h. During the reaction, the toluene-water azeotrope was distilled out of the system and collected in the Dean–Stark trap. After cooling to room temperature, the viscous solution was carefully poured into an excess of ethanol to yield a white resin. The obtained PI-I_a_ resin was collected and dried at 100 °C in vacuum for 24 h. Yield: 41.2 g (96%). ^1^H-NMR (DMSO-*d*_6_, ppm): 10.07 (s, 1H), 8.08–8.05 (d, 2H), 7.47–7.42 (m, 3H), 7.14–7.10 (d, 2H), 3.25 (m, 4H), 2.33–2.29 (m, 2H), 2.25–2.23 (m, 3H), and 2.16–2.11 (m, 2H).

The other PI resins, including PI-I_b_, PI-II_a_, and PI-II_b_ were prepared according to a similar procedure as mentioned above.

PI-I_b_. Yield: 43.2 g (96%). ^1^H-NMR (DMSO-*d*_6_, ppm): 10.32 (s, 1H), 8.11 (s, 2H), 7.73 (m, 1H), 7.63 (m, 1H), 7.49 (m, 2H), 7.32 (m, 1H), 3.27 (m, 4H), 2.30–2.26 (m, 2H), and 2.09–2.03 (m, 2H).

PI-II_a_. Yield: 49.1 g (96%). ^1^H-NMR (DMSO-*d*_6_, ppm): 10.08 (s, 1H), 8.10–8.07 (d, 2H), 7.73–7.36 (m, 3H), 7.22–7.13 (m, 2H), 3.27 (m, 4H), 3.04–3.02 (m, 4H), 2.27 (m, 3H), 2.18–2.08 (m, 4H), 1.65 (m, 4H), 1.31–1.20 (m, 4H), and 1.06–1.03 (m, 2H).

PI-II_b_. Yield: 51.1 g (96%). ^1^H-NMR (DMSO-*d*_6_, ppm): 10.30 (s, 1H), 8.12–8.09 (d, 2H), 7.74–7.71 (m, 1H), 7.62–7.59 (m, 1H), 7.50 (m, 2H), 7.36 (m, 1H), 3.26 (m, 4H), 3.06–3.03 (m, 4H), 2.17–2.09 (m, 4H), 1.68–1.50 (m, 4H), and 1.32–1.04 (m, 6H).

The fully dried PI-I_a_ resin was dissolved in newly distilled DMAc at room temperature with the solid contents (*S*_c_) in the range of 5–40 wt % to afford a series of PI-I_a_ varnishes. The absolute viscosities of the obtained varnishes were measured and the viscosity-*S*_c_ relationship was established. Then, PI-I_a_ varnish with a solid content of 35 wt % was filtered through a 0.45 μm Teflon syringe filter to remove any impurities. Then, the solution was spin-coated on a clean silicon wafer or quartz substrate. The thickness of the PI-I_a_ film for various measurements was controlled by regulating the spinning rate. PI-I_a_ films with the thicknesses ranged from 10–100 μm were obtained by thermally baking the PI solution in flowing nitrogen according to the following heating procedure: 80 °C/3 h, 150 °C/1 h, 180 °C/1 h, 200 °C/1 h, and 250 °C/1 h.

The other PI films, including PI-I_b_, PI-II_a_, and PI-II_b_ were prepared according to a similar procedure as mentioned above.

## 3. Results and Discussion

### 3.1. PI Synthesis and Film Preparation

**Figure 1:**
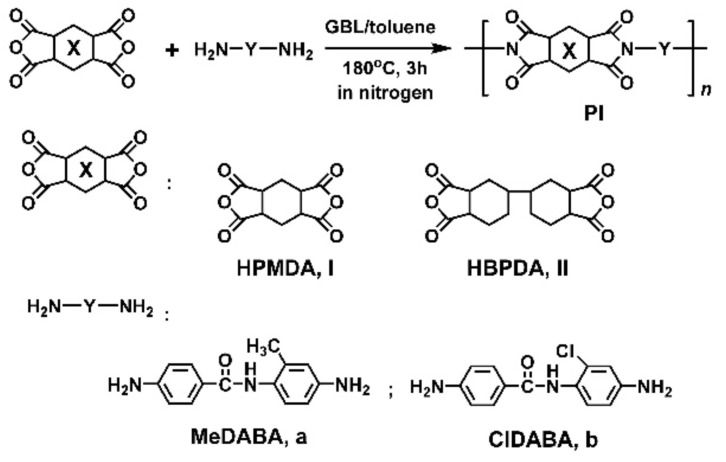
Preparation of semi-alicyclic amide-bridged PIs.

**Figure 2:**
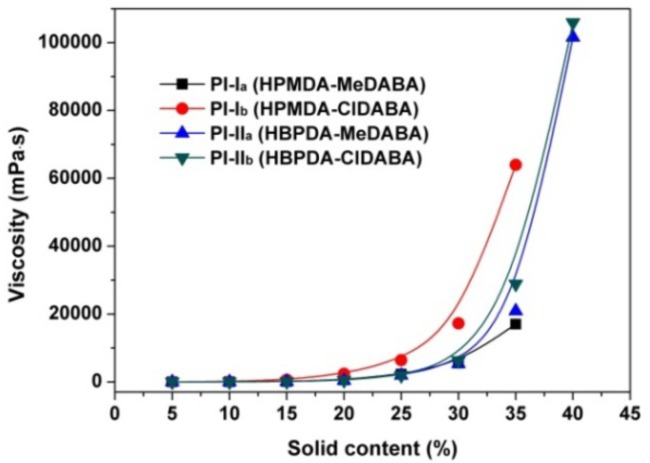
Viscosity–solid content relationship of the PI solutions (Solvent: DMAc).

The methyl-substituted amide-bridged aromatic diamine MeDABA was synthesized first and then polymerized with the alicyclic dianhydrides via a high-temperature polycondensation procedure. Another commercially available chlorine-substituted amide-containing ClDABA diamine was also polymerized to afford the PIs for references. The synthesis pathway was shown in Figure 1. Homogeneous polymerization solution was obtained for both of the two systems, indicating good solubility of the PIs in the solvent. Under the same condition, the polymerization reaction of HPMDA or HBPDA with the unsubstituted diamine, 4,4’-diaminobenzanilide (DABA) only provide gelled solution. The inherent viscosities, molecular weights, and solubility of the derived PI resins are tabulated in Table 1. The PI resins possessed moderate to high molecular weights with numerical molecular weights (*M*_n_s) in the range of 22.1 × 10^3^–37.6 × 10^3^ g/mol, indicating the good reactivity of the amide-bridged diamines. The PI resins showed good solubility in polar aprotic solvents, such as NMP, DMAc, and GBL. Basically, the HBPDA-PIs showed superior solubility compared to the HPMDA-PI analogues, which were soluble in cyclopentanone and partially soluble in tetrahydrofuran (THF). This is mainly attributed to the loose molecular packing caused by the flexible dicyclohexane units, which is quite beneficial for the penetration of organic solvents. In addition, the difference in the effects of the methyl or chlorine substituents in the diamines on the solubility of the PI resins is not significant when the same dianhydride was used. The PI resin prepared from the two diamine monomers showed similar dissolution behavior in the organic solvents. Although the PI resins showed similar dissolution behavior in the tested solvents, the degree of solubility was quite different. Figure 2 shows the quantitative change of the absolute viscosities of the PI solutions with the solid contents (*S*_c_) of the resins in DMAc. It can be clearly observed that the viscosities of the PI solutions smoothly increase with the *S*_c_ values of the solutions. When the *S*_c_ values are higher than 30 wt %, the viscosities increased markedly. At the same *S*_c_, such as 35 wt %, the viscosities increased according to the sequence of PI-I_a_ (17,010 mPa s) < PI-II_a_ (20,940 mPa s) < PI-II_b_ (28,860 mPa s) < PI-I_b_ (63,980 mPa s). This trend is in good agreement with that of the *M*_n_ values of the resins.

**Figure 3:**
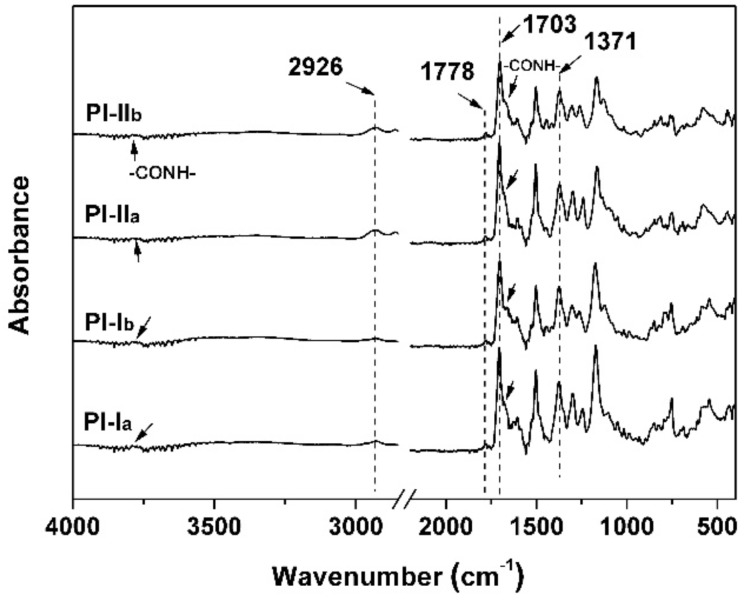
ATR-FTIR spectra of PI resins.

The chemical structures of the PI resins were then confirmed by the ATR-FTIR and NMR measurements. Figure 3 shows the ATR-FTIR spectra of the PI films. All the characteristic absorptions of imide rings, including the peaks at 1778 cm^−1^ induced by the asymmetrical carbonyl stretching vibrations, the peaks at 1703 cm^−1^ assigned to the symmetrical carbonyl stretching vibrations, and the peaks at 1371 cm^−1^ for the C–N stretching vibrations were clearly observed. In addition, the characteristic absorptions of –CONH– units consisted of the N-H part at 3770 cm^−1^ and the C=O part at 1672 cm^−1^ were also detected. Meanwhile, the stretching vibrations at 2926 cm^−1^ attributed to the –CH and –CH_2_ groups in cyclohexane units in the dianhydride moieties and –CH_3_ groups in the diamine units were all detected.

**Figure 4:**
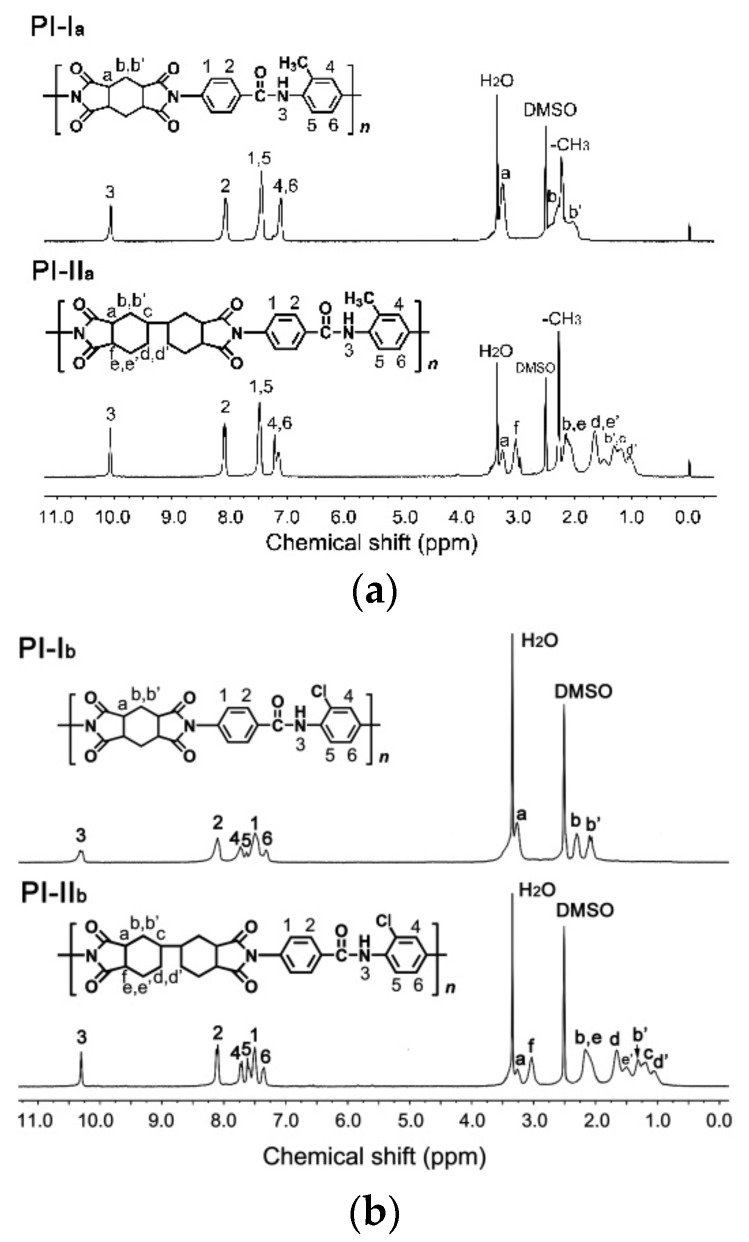
^1^H-NMR spectra of PI resins. (**a**) MeDABA-PIs; (**b**) ClDABA-PIs.

Figure 4 depicts the ^1^H-NMR spectra of the PI resins purified by several dissolution–precipitation circulations followed by dried at 100 °C in vacuum together with the assignments of individual proton. All the resins were soluble in the NMR solvent, DMSO-*d*_6_ and clear absorptions for hydrogen protons were recorded. The spectra shown in Figure 4 indicate that all hydrogen peaks agreed well with the proposed structures. The protons in the amide (–CONH–) moieties (H_3_) appeared at the farthest downfield (chemical shift: ~10.0 ppm) in the spectra, owing to the strong electron-withdrawing carbonyl group. The absorptions of the protons in the aromatic phenyl rings (H_1_, H_2_, H_4_, H_5_, and H_6_) appeared at the second farthest downfield in the spectra. The protons *ortho*-substituted to the electron-withdrawing carbonyl group in the amide linkages (H_2_) showed higher chemical shift values compared to the other protons in the aromatic phenyl rings. The signals at the upfield areas are attributed to the absorptions of cyclohexane ring hydrogen protons in the dianhydride units. The protons *ortho-*substituted to the electron-withdrawing carbonyl group in the anhydride units, that is the H_a_ protons for PI-I_a_ and PI-I_b_ and H_a_ and H_f_ protons for PI-II_a_ and PI-II_b_, showed relatively higher chemical shift values compared to the other protons in the alicyclic rings. For the HBPDA-PIs, much more absorptions were detected due to the different chemical environments of the proton pairs in the cyclohexane rings, including H_b,b’_, H_d,d’_, and H_e,e’_. In summary, the chemical structural features revealed by the ATR-FTIR and ^1^H-NMR measurements indicate that we successfully synthesized the PI resins with the anticipated structures.

**Figure 5:**
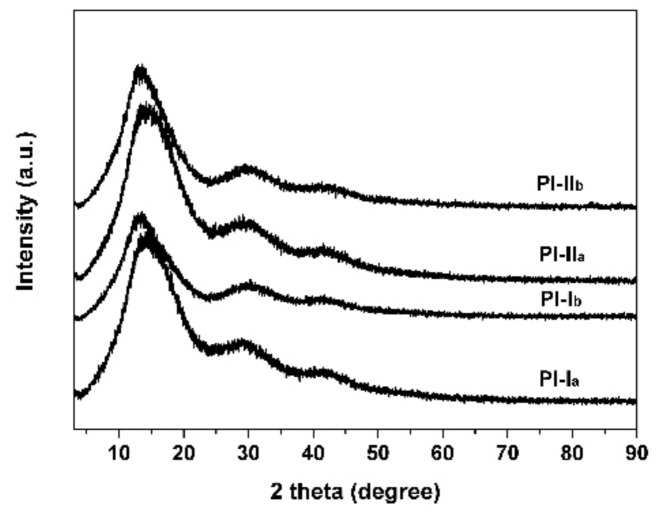
XRD spectra of PI films.

The microscopic crystallinity of the PI resins was detected by XRD measurements and the results are shown in Figure 5. Generally, the rigid-rod amide bonds are easily to endow the polymers crystalline features; thus the polymer films containing amide bonds, such as polyamide films, generally show semi-crystallization properties. However, in the current work, according to the XRD plots shown in Figure 5, the PI films exhibited amorphous nature and no sharp crystalline peaks were detected for all of the films. This is mainly attributed to the loose molecular packing of the alicyclic cyclohexane units in the dianhydride moiety. Meanwhile, the methyl or chlorine substituents in the diamine moiety might destroy the crystallinity of the PI molecular chains. The amorphous nature of the current PI resins is quite beneficial for improving the solubility in organic solvents and increasing the optical transparency of the derived films.

### 3.2. Optical Properties

**Figure 6:**
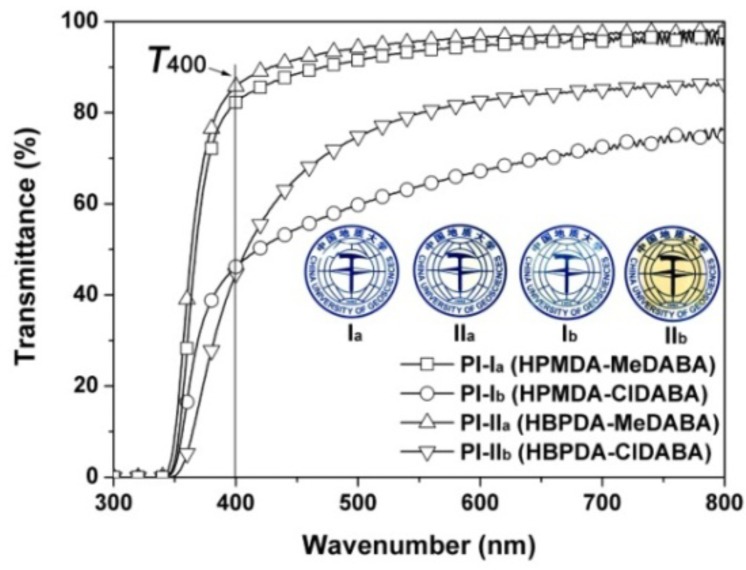
UV–Vis spectra of PI films. Insert: appearance of PI films.

**Figure 7:**
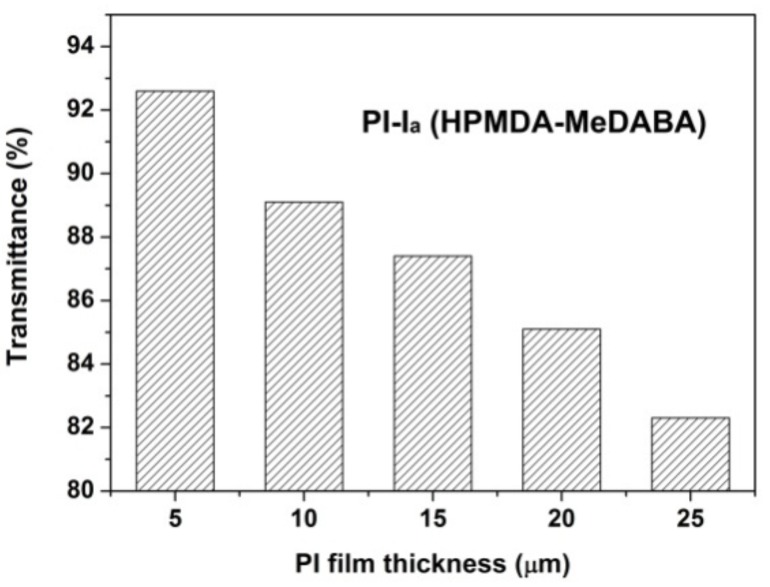
Thickness–transmittance relationship of PI-I_a_ film.

The effects of the chemical structures of the PI films on their optical properties were investigated by the optical transmittance and color measurements. The flexible and tough PI films with the thickness in the range of 10–50 μm were successfully prepared from the PI/DMAc solutions with different solid contents. The ultraviolet-visible (UV–Vis) spectra of the PI films at a thickness of 25 μm were measured and shown in Figure 6 together with the appearance of the PI films. The optical data were listed in Table 2. Basically, for the same dianhydride monomer, the PI films derived from MeDABA (PI-I_a_ and PI-II_a_) showed much higher optical transmittances than those of the ClDABA analogues (PI-I_b_ and PI-II_b_). For instance, PI-I_a_ (HPMDA-MeDABA) film has a cutoff wavelength (λ) of 338 nm and an optical transmittance of 82.3% at the wavelength of 400 nm, which is 9 nm lower and 36.1% higher than those of the PI-I_b_ (HPMDA-ClDABA) film, respectively. The thickness-transmittance relationship of PI-I_a_ film at the wavelength of 400 nm is shown in Figure 7. The optical transmittance of the PI film decreased with the increasing of the thickness of the film. The film has a maximum transmittance over 92% when its thickness is 5 μm.

**Figure 8:**
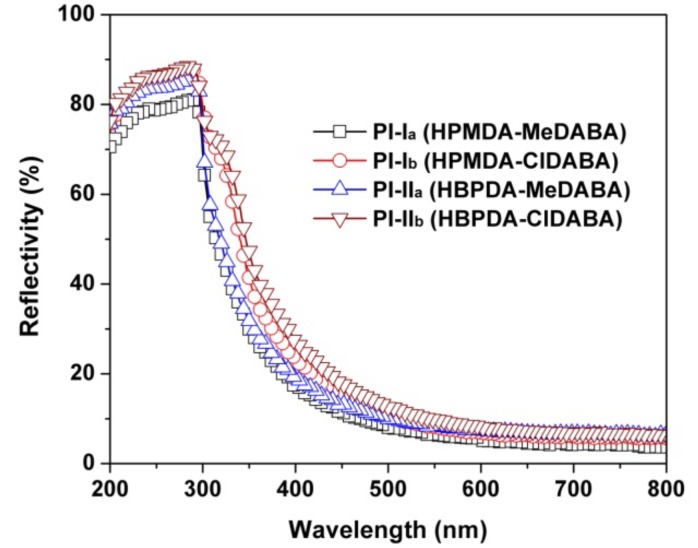
UV–Vis reflectivity spectra of PI films.

The optical reflectivity behaviors of the PI films were also investigated and the results are presented in Figure 8. The reflectance behaviors of the PI films were investigated by measuring the reflectivity values of the PI films. The PI films showing better optical transmittances in Figure 6 revealed lower reflectivity values in Figure 8. For example, PI-I_a_ (HPMDA-MeDABA) film shows a reflectivity value of 17.4% at the wavelength of 400 nm, which is comparable to that of PI-II_a_ (HBPDA-MeDABA) (19.3%) and apparently lower than those of the PI-I_b_ (HPMDA-ClDABA) (reflectivity: 23.1%) and PI-II_b_ (HBPDA-ClDABA) (reflectivity: 27.4%).

**Figure 9:**
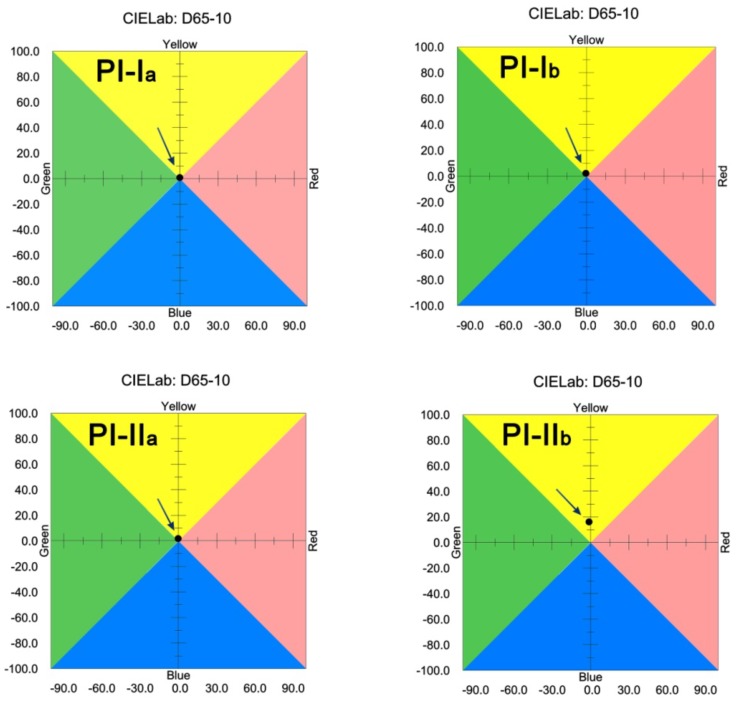
CIE Lab color parameters of PI films.

The inferior optical transparency of the ClDABA-PIs could be interpreted by the CIE Lab color parameters measurements, as shown in Figure 9 and Table 2. It can be seen that the ClDABA-PI films showed much higher haze values than those of the MeDABA-PI counterparts. For instance, PI-I_b_ showed a haze value of 11.05%, which was 8.9% higher than that of PI-I_a_ film. The higher haze values of the ClDABA-PI films indicated the existence of microcrystalline regions in the films, although they were not observed by the XRD measurements. On the other hand, the worse optical transparency of the ClDABA-PIs might also be contributed to the larger bond polarizability coefficients of C-Cl bond compared to that of C-C bond [33]. It has been well known that introduction of groups with low polarizability is helpful to increasing the optical transparency of the derived films [34]. PI-I_a_ and PI-II_a_ films showed the best optical properties among the films with the *T*_400_ values higher than 82%, yellow indices (b*) around 2.0, and haze values lower than 2.2%. On the contrary, PI-II_b_ film showed the worst optical properties with a pale-brown appearance (b* = 16.05), as illustrated by the insert in Figure 6.

### 3.3. Thermal Properties

Good thermal stabilities, including high thermal decomposition temperatures, high glass transition temperatures (*T*_g_s), and high dimensional stability at elevated temperatures are critical for the practical applications of CPI films, especially for the semi-alicyclic ones. The semi-alicyclic CPI films usually exhibit inferior thermal properties compared to the fluoro-containing wholly aromatic counterparts due to the thermally unstable nature of the alicyclic groups. The inferior thermal properties of the semi-alicyclic CPI films can usually remedied by introduction of rigid chemical bonds in the diamine moiety. In the current work, the rigid-rod amide linkages were introduced into the molecular structures of the semi-alicyclic CPI films via the diamine monomer so as to improve the thermal properties of the polymers. The thermal stabilities of the PI films were investigated by TGA, DSC, and TMA measurements, respectively. The thermal data are summarized in Table 2.

**Figure 10:**
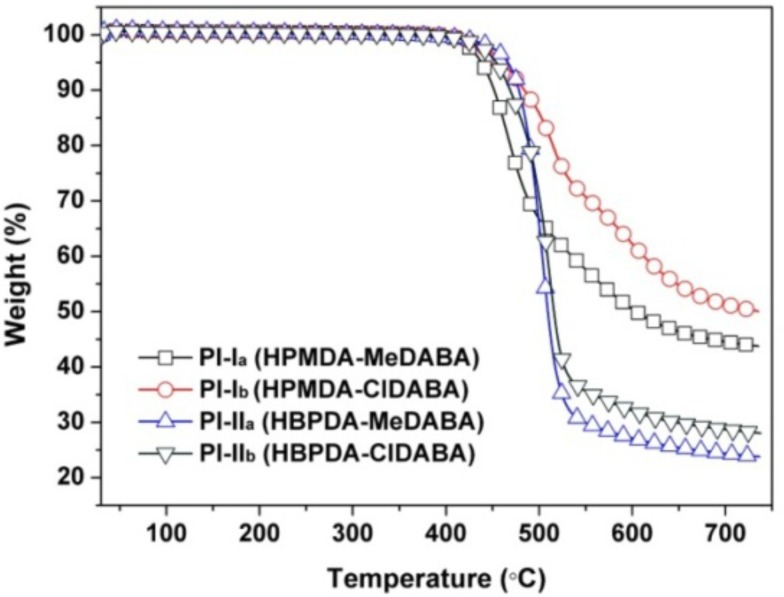
TGA curves of PI films in nitrogen.

Figure 10 shows the TGA plots of the PI films measured in the range of 50 to 730 °C in nitrogen. Basically, the current PI films exhibited good thermal stability up to 400 °C, after which they decomposed rapidly and lost nearly 50–80% of their original weights at 730 °C. In terms of the residual weight ratios of the PI films at 730 °C (*R*_w730_), for the same diamine monomer, the PI films derived from HPMDA (PI-I_a_ and PI-I_b_) showed better thermal stability than those from the HBPDA (PI-II_a_ and PI-II_b_). For instance, PI-I_a_ (HPMDA-MeDABA) film has an *R*_w730_ value of 43.9%, which was 20.0% higher than that of the PI-II_a_ (HBPDA-MeDABA) film. On the other hand, for the same dianhydride monomer, the PI films derived from ClDABA (PI-I_b_ and PI-II_b_) showed better thermal properties than those from the MeDABA (PI-I_a_ and PI-II_a_). For instance, PI-I_b_ (HPMDA-ClDABA) film has an *R*_w730_ value of 50.2%, which was 6.3% higher than that of the PI-I_a_ (HPMDA-MeDABA) film. These phenomena could be ascribed to the inferior thermal resistance of the HBPDA and MeDABA.

**Figure 11:**
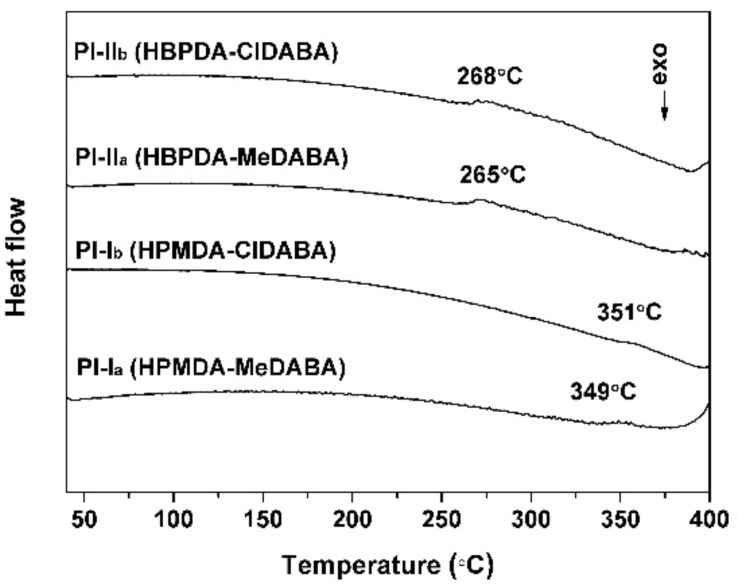
DSC curves of PI films.

The glass transition temperatures (*T*_g_) of the PI films were determined by the DSC measurements, as shown in Figure 11. During the heating, the PI films exhibited stable glassy state before 260 °C for HBPDA-PI films and 345 °C for HPMDA-PI films. After the temperatures, clear glass transition behaviors were observed for all of the PI films. Comparatively, the HPMDA-PIs exhibited much higher *T*_g_ values than those of the HBPDA-PIs due to the relatively higher molecular chains rigidity of the former. With respect to the effects of diamine monomers, the PIs derived from ClDABA showed slightly higher *T*_g_ values than those of the MeDABA-PIs due to the similar structural features of chlorine and methyl substituents. As it turns out, the current PI films, especially the HPMDA-PIs exhibit excellent thermal stability and can meet most of the thermal requirements for optoelectronic applications. Introduction of the rigid-rod amide inevitably contributes to the improvement of the thermal stability of the PI films, which might form strong intermolecular hydrogen bonds and effectively prohibit the free rotation of the molecular chains at elevated temperatures.

**Figure 12:**
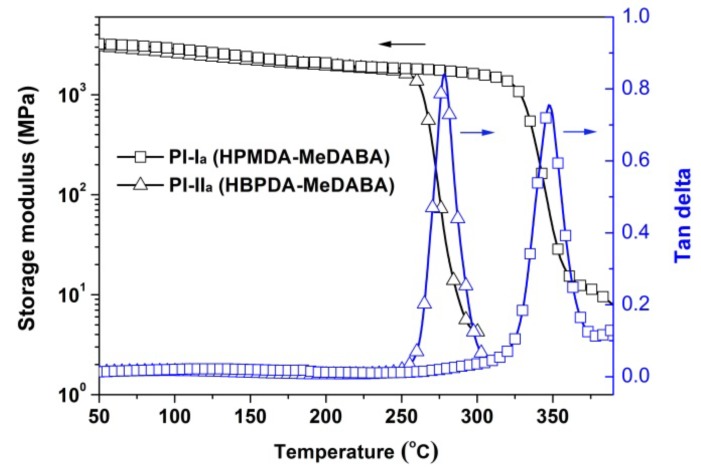
DMA curves of MeDABA-PI films.

The good thermal stability of the amide-bridged PI films can also be reflected by the modulus change, as illustrated by the DMA measurements of MeDABA-PI films in Figure 12. The PI films showed stable storage modulus in the range of 10^3^~10^4^ MPa before 250 °C for PI-II_a_ and 300 °C for PI-I_a_. The peak values of the tan delta measurements in DMA revealed the *T*_g_ values of the PI films. The *T*_g_ values of the polymers are 347 °C for PI-I_a_ and 279 °C for PI-II_a_, respectively.

At last, the effects of the rigid-rod amide linkages on the high-temperature dimensional stability of the PI films were investigated by the TMA measurements. From the viewpoint of structural characteristics, polymer films with low linear coefficients of thermal expansion (low-CTE) usually contain rigid or highly conjugated aromatic groups in the molecular structures. Thus, the semi-alicyclic PI films with the flexible alicyclic moieties in the structures are expected to possess higher CTE values than the wholly aromatic analogues. It has been established in the literature that it is quite difficult to reduce the CTEs values of the semi-alicyclic PIs via the structural modification of the alicyclic dianhydride part. Even if rigid chemical bonds—such as amide bond, ester bond, and so on—are introduced into the alicyclic dianhydride unit, it is still difficult to reduce the CTE values of the semi-alicyclic PI films to a great extent. For example, the semi-alicyclic PI films derived from the ester-linked alicyclic dianhydride and aromatic diamines exhibited CTE values higher than 64 × 10^−6^/K in the temperature range of 100–150 °C [26]. Even the semi-alicyclic PI films derived from the rigid alicyclic dianhydride, such as norbornane-2-spiro-α-cyclopentanone-α‘-spiro-2’’-norbornane-5,5’’,6,6’’-tetracarboxylic dianhydride (CpODA) and the flexible aromatic diamines, such as 4,4’-oxydianiline (4,4’-ODA) and 3,4’-oxydianiline (3,4’-ODA) showed high CTE values of 49 × 10^−6^/K for the former and 57 × 10^−6^/K for the latter in the temperature range of 100~200 °C [35]. On the contrary, the introduction of rigid components in the diamine part has been proven to be an efficient pathway decreasing the CTE values of the semi-alicyclic PI films. For example, for the same alicyclic dianhydride CpODA, the PI film derived from the rigid 4,4’-diamino-benzanilide (4,4’-DABA) showed a low CTE value of 15 × 10^−6^/K [36]. However, the PI (CpODA-4,4’-DABA) was not soluble in organic solvents and could only be processed via the thermal imidization procedure.

The homo-polymerized HPMDA-PI films usually exhibited CTE values in the range of (40~60) × 10^−6^/K [37]. The main purpose of the current work is to reduce the CTE values of the HPMDA-PI films to a level below 35 × 10^−6^/K while maintaining their intrinsic property merits, such as good solubility in organic solvents for the resins and acceptable optical transparency for the films so as to meet the general optoelectronic application requirements. On the other hand, the derived low-CTE HPMDA-PIs might be used as the matrix to be combined with the inorganic additives at a low loading level for further decreasing the CTE values without scarifying the optical properties of the composite films.

**Figure 13:**
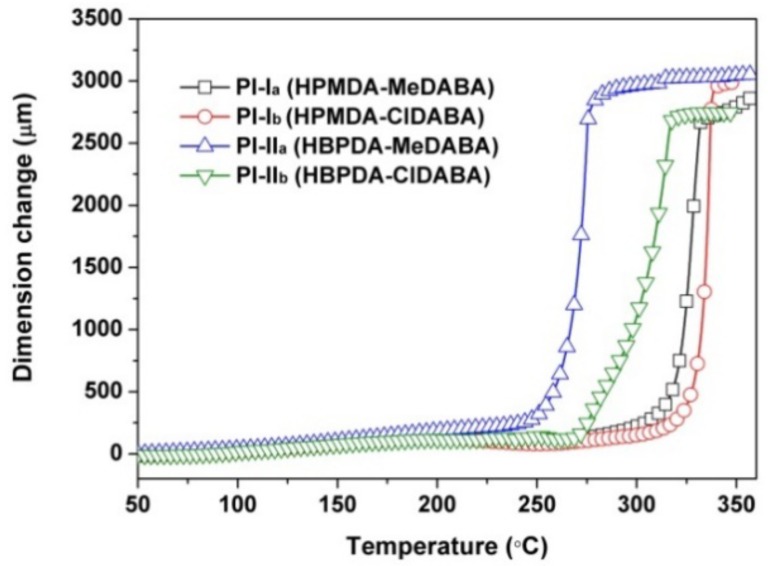
TMA curves of PI films.

Figure 13 depicts the TMA plots of the PI films and the CTE values are tabulated in Table 2. As expected, the HBPDA-PIs (PI-II_a_ and PI-II_b_) showed moderate CTE values of 55.3 × 10^−6^/K for PI-II_a_ and 53.2 × 10^−6^/K for PI-II_b_ in the temperature range of 50–250 °C, even if the rigid amide-containing diamines were used. However, for the HPMDA-PI films, satisfactory results were obtained. PI-I_a_ (HPMDA-MeDABA) and PI-I_b_ (HPMDA-ClDABA) films exhibited obviously reduced CTE values of 33.4 × 10^−6^/K and 27.7 × 10^−6^/K, respectively, achieving the research target of the current work. Apparently, this achievement was attributed to the joint action of the rigid amide bridges in the diamine moiety and cyclohexane ring in the dianhydride unit. In both circumstances, the CTE values of PI films prepared from ClDABA and the alicyclic dianhydrides were all slightly lower than those derived from MeDABA.

## 4. Conclusions

The inherently high CTE values of semi-alicyclic PI films greatly prohibit the wide applications in advanced optoelectronic fabrications. Although various methods for reducing the CTEs of semi-alicyclic PI films have been reported in the literature, most of the results are not very satisfactory. In this study, the rigid amide bond was introduced into the molecular structures of the diamine monomers so as to reduce the CTE values of the derived semi-alicyclic PI films. The experimental results revealed the efficiency of this methodology. The PI-I_a_ film derived from HPMDA and MeDABA showed the best combined properties, including a CTE value of 33.4 × 10^−6^/K in the temperature range of 50–250 °C, a *T*_5%_ value of 438.1 °C, a *T*_g_ value of 349 °C, a *T*_400_ value of 82.3% at the wavelength of 400 nm with a thickness of 25 µm, a yellow index (b^*^) of 2.18, and a haze value of 2.15%. The good combined properties make the PI-I_a_ film good candidates for advanced optoelectronic applications.

## Figures and Tables

**Table 1:** Inherent viscosities, molecular weights, and solubility of SPI resins.

PI	[*η*]_inh_^a^ (dL/g)	Molecular Weight ^b^		Solubility ^c^					
		*M*_n_ (× 10^3^ g/mol)	*M*_w_ (× 10^3^ g/mol)	PDI	NMP	DMAc	GBL	CPA	THF
PI-I_a_	1.03	22.1	39.6	1.79	++	++	++	+	−
PI-I_b_	1.16	37.6	67.2	1.29	++	++	++	+	−
PI-II_a_	0.92	26.3	46.7	1.78	++	++	++	++	+
PI-II_b_	0.97	29.8	54.6	1.83	++	++	++	++	+

^a^ Inherent viscosities measured with a 0.5 g/dL PI solution in NMP at 25 °C; ^b^ *M*_n_: number average molecular weight; *M*_w_: weight average molecular weight; PDI: polydispersity index, PDI = *M*_w_/*M*_n_; ^c^ ++: Soluble; +: partially soluble; −: insoluble. GBL: γ-butyrolactone; CPA: cyclopentanone; THF: tetrahydrofuran.

**Table 2:** Optical and thermal properties of PI films

PI	Optical Properties ^a^							Thermal Properties ^b^		
	λ (nm)	*T*_400_ (%)	L*	a*	b*	Haze (%)	*T*_g_ (°C)	*T*_5%_ (°C)	*R*_w730_ (%)	CTE (× 10^−^^6^/K)
PI-I_a_	338	82.3	94.91	−0.13	2.18	2.15	349	438.1	43.9	33.4
PI-I_b_	347	46.2	94.28	−0.42	2.54	11.05	351	466.8	50.2	27.7
PI-II_a_	341	85.8	95.41	−0.17	1.92	1.07	265	466.3	23.9	55.3
PI-II_b_	342	44.7	91.16	−1.20	16.05	10.41	268	453.9	28.1	53.2

^a^ λ: Cutoff wavelength; *T*_400_: Transmittance at the wavelength of 400 nm with a thickness of 50 µm; L*, a*, b*, see Measurements part. ^b^ *T*_g_: Glass transition temperature; *T*_5%_: Temperatures at 5% weight loss; *R*_w7__30_: Residual weight ratio at 730 °C in nitrogen; CTE: linear coefficient of thermal expansion in the range of 50–250 °C.
